# A novel and practical asymmetric synthesis of eptazocine hydrobromide

**DOI:** 10.3762/bjoc.14.209

**Published:** 2018-09-06

**Authors:** Ruipeng Li, Zhenren Liu, Liang Chen, Jing Pan, Kuaile Lin, Weicheng Zhou

**Affiliations:** 1 State Key Lab of New Drug & Pharmaceutical Process, Shanghai Key Lab of Anti-Infectives, Shanghai Institute of Pharmaceutical Industry, China State Institute of Pharmaceutical Industry, No. 285, Gebaini Rd., Shanghai 201203, P. R. of China

**Keywords:** alkylation, asymmetric catalysis, eptazocine, Mannich cyclization

## Abstract

In order to prepare eptazocine hydrobromide effectively, a novel, mild and practical asymmetric process was developed starting from 1-methyl-7-methoxy-2-tetralone under the catalysis of *N*-(*p*-trifluoromethylbenzyl)cinchonidinium bromide. The reaction conditions were optimized to obtain the product in excellent overall yield and purity.

## Introduction

**Scheme 1:**
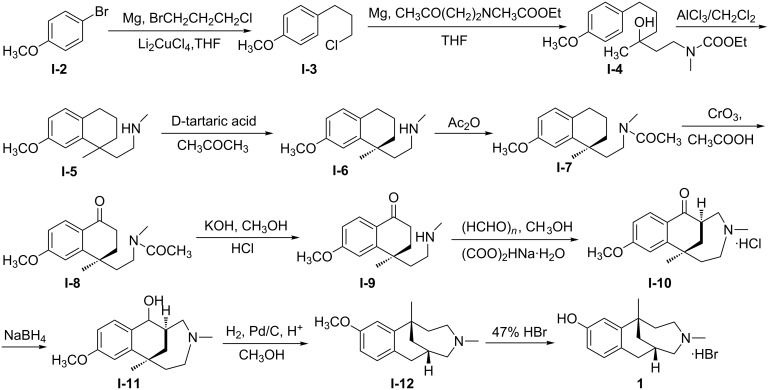
Commercial process for the synthesis of **1**.

Eptazocine hydrobromide (**1**, Scheme 1), (1*S*,6*S*)-1,4-dimethyl-2,3,4,5,6,7-hexahydro-1*H*-1,6-methanobenzo[e]azonine-10-ol hydrobromide, developed by Nihon lyakuhin Kogyo Co., Ltd., was extensively used as a narcotic-antagonizing analgesic for relieving post-operative pains, pains from cancer, etc. [1–2]. The commercial synthetic route to **1**, shown in Scheme 1, involved the traditional resolution with concomitant discard of 50% in mass of the unwanted isomer [3–6]. Other asymmetric syntheses of **1** have been reported in the literature. These relied either on organometallic catalysis [7], asymmetric tandem addition to chiral tetrahydronaphthalenes [8], bioenzymatic steps [9] or diastereoselective Evans alkylation from oxazolidinone and methallyl iodide [10]. However, these methods were undermined by expensive reagents, poor yields, harsh reaction conditions, or complex synthetic procedures.

**Scheme 2:**
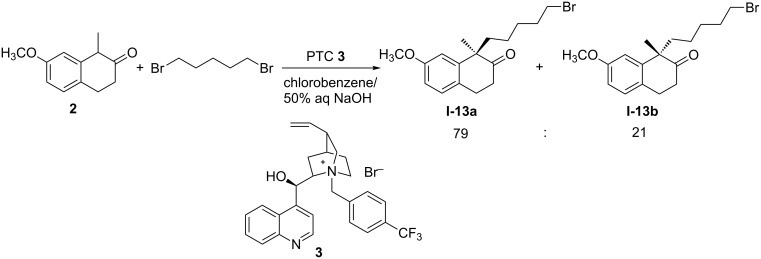
Previous work about asymmetric synthesis of **I-13a**.

Phase-transfer asymmetric catalysis using cinchona alkaloid-derived quaternary ammonium salts is a practical method in organic synthesis [11–13]. In our prior publication [14], this method was selected as the technology for the development of a process to prepare (*R*)-(+)-1-(5’-bromopentyl)-1-methyl-7-methoxy-2-tetralone, a key intermediate of dezocine, and *N*-(*p*-trifluoromethylbenzyl)cinchonidinium bromide (**3**) among 17 cinchona alkaloid-derived catalysts was identified as the best one for asymmetric alkylation of 1-methyl-7-methoxy-2-tetralone (**2**) with 1,5-dibromopentane. The products **I-13a** and **I-13b** were isolated in a ratio of 79:21 and in 77.8% yield (Scheme 2). Encouraged by the previous studies and relevant works [15–16], we are engaged in the development of a concise and efficient asymmetric synthetic route for eptazocine hydrobromide (**1**), with the same material and catalyst.

## Results and Discussion

**Scheme 3:**
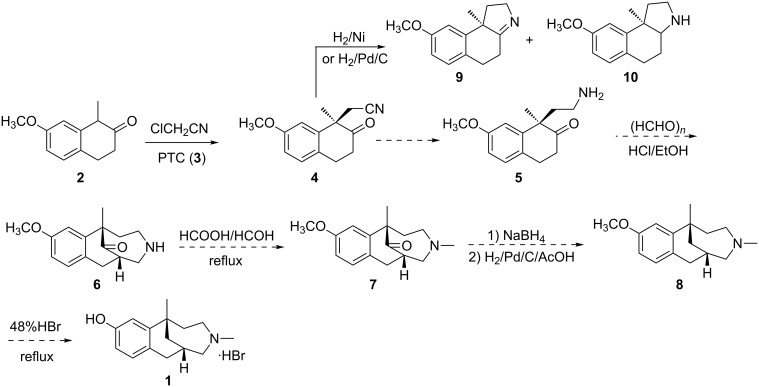
Asymmetric synthesis of **1**.

Herein, we developed a new, practical and resolution-free preparation of **1** (Scheme 3) using the asymmetric alkylation of **2** in the presence of catalyst PTC (**3**).

The designed synthesis is shown in Scheme 3. Two key reactions are included in this novel strategy: 1. the enantioselective alkylation of 1-methyl-7-methoxy-2-tetralone (**2**) with chloroacetonitrile generate **4** through the use of a phase-transfer catalyst; and 2. the Mannich reaction to construct the tricyclic framework of **6**. The study started with the alkylation under catalysis of **3** in a two-phase system of chlorobenzene and 50% aqueous NaOH solution based on our previous report [14]. When the reaction was run at 0–5 °C, an excellent yield (70%) and enantiomeric ratio (**4**/**4’** 81:19) were achieved (Table 1, entry 2). Subsequently, the yield was improved when the concentration of NaOH decreased from 50% to 30% (Table 1, entry 3). However, further decreasing the concentration of the base made the reaction less productive (Table 1, entry 4). For optimization of the amount of chloroacetonitrile, it was found that 2 equiv were adequate for the reaction (Table 1, entries 5 and 6). As far as the substrate concentration was concerned, an increasing substrate concentration resulted in a slight decrease of the enantiomeric ratio (Table 1, entry 7). Finally, the key reaction was scaled up (60 g of **2**) according to the conditions in entry 5, and similar results were obtained (Table 1, entry 8). After the screening of many solvents, the pure isomer **4** was isolated from the crude reaction mixture through crystallization from ethyl acetate with 48% yield and >99% chiral purity.

**Table 1:** Optimization of asymmetric alkylation.^a^

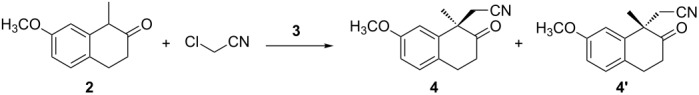							

entry	ClCH_2_CN (equiv)	conc. (mol/L)	temperature (°C)	base	reaction time (h)	yield^b^	**4**:**4’**^c^

1	3	0.07	15–25	50% aq NaOH	2	trace	–
2	3	0.07	0–5	50% aq NaOH	1.5	70.0%	81:19
3	3	0.07	0–5	30% aq NaOH	2	78.6%	80:20
4	3	0.07	0–5	10% aq NaOH	4	trace	–
5	2	0.07	0–5	30% aq NaOH	2	78.4%	80:20
6	1.5	0.07	0–5	30% aq NaOH	6	61.0%^d^	79:21
7	2	0.13	0–5	30% aq NaOH	1.5	79.2%	73:27
8^e^	2	0.07	0–5	30% aq NaOH	2	78.8%	80:20

^a^The reaction was performed with **2** (2.0 g), ClCH_2_CN and aq NaOH in chlorobenzene in the presence of 10 mol % of **3** under N_2_ atmosphere. ^b^Isolated yield including **4** and **4’**. ^c^Determined by HPLC at 210 nm using Chiralpak AY-H as a chiral column with hexane/isopropyl alcohol 50:50 as the eluent. ^d^The reaction was incomplete, since compound **2** was detected by TLC even after longer reaction time. ^e^60 g of **2** was used.

**Scheme 4:**
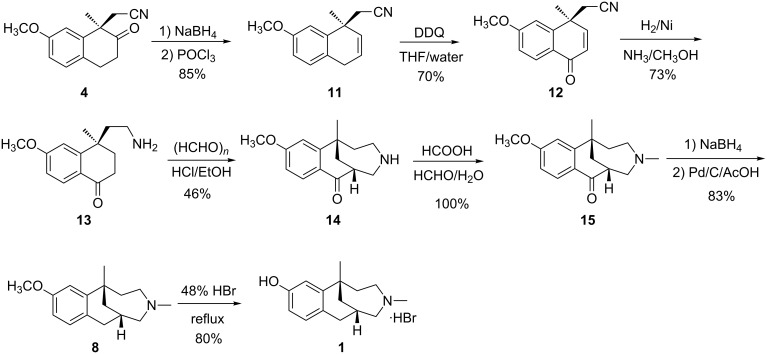
The second strategy for the asymmetric synthesis of **1**.

With purified **4** in hand, the next step was the reduction. When compound **4** was reduced by catalytic hydrogenation at 0.4 MPa in the presence of Raney-Ni as catalyst in NH_3_/CH_3_OH, compounds **9** and **10** were formed as the main products instead of the expected compound **5**. When the solvent NH_3_/CH_3_OH was replaced with HOAc/H_2_O (2:1) or Pd/C was selected as a catalyst, the reaction gave a similar result as with Raney-Ni as catalyst in NH_3_/CH_3_OH. It seems that compound **5** easily reacted with the carbonyl group to form the five-membered ring. Since the carbonyl group is necessary for the following Mannich reaction, a second strategy was proposed as illustrated in Scheme 4.

Tetralone **4** was converted to dihydronaphthalene **11** by reduction with NaBH_4_ in methanol, followed by dehydration with POCl_3_/pyridine at reflux (85% yield). In order to get ketone **12**, a series of oxidation conditions was tested (Table 2). In general, CrO_3_ was commonly used for this reaction [17–18], but, when the reaction was run with CrO_3_ in acetic acid/water (Table 2, entry 1), a low yield (33%) was obtained. However, the reaction with CrO_3_/acetic anhydride/acetic acid in dichloromethane gave a slightly improved yield (Table 2, entry 3). When other oxidants, such as selenium oxide and manganese dioxide, were used, even at reflux temperature, no reaction took place (Table 2, entries 4 and 5). Owing to the concern of heavy metal pollution from the metal oxidant, organic oxidants were tested. Fortunately, DDQ in dioxane could oxidize compound **11** to **12**, although the yield was 36% (Table 2, entry 6). The data of solvent screening are included in entries 6–10 in Table 2. It seems that water was crucial to improve the yield, because entries 8 and 9 gave the best results (69–73%). Finally, the oxidation was scaled up (30 g of **11**) using the conditions in entry 9 and a similar yield was obtained (Table 2, entry 11).

**Table 2:** Optimization of oxidation conditions.

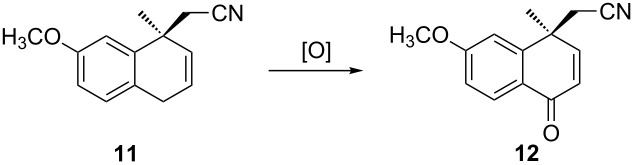				

entry	oxidant	solvent	reaction time (h)	yield

1	CrO_3_	CH_3_COOH/water	1.5	33.2%
2	CrO_3_	DCM/CH_3_COOH	2	30.1%
3	CrO_3_	DCM/(CH_3_CO)_2_/CH_3_COOH	2	45.7%
4	SeO_2_	dioxane	6	no reaction
5	MnO_2_	dioxane	6	no reaction
6	DDQ	dioxane	2	35.5%
7	DDQ	DCM	2.5	trace
8	DDQ	dioxane/DCM/water	1.5	73.5%
9	DDQ	THF/water	2	69.3%
10	DDQ	THF	2	25.2%
11	DDQ	THF/water	2	70.1%

When cyano compound **12** was reduced by hydrogenation in the presence of Raney-Ni as catalyst in NH_3_/CH_3_OH, the proposed primary amine **13** was obtained in 73% yield.

Then, for the Mannich cyclization, when **13** was reacted with paraformaldehyde in HCl/MeOH at reflux, compound **14** was obtained in 26% yield (Table 3, entry 1). A better yield (45–46%, Table 3, entries 2 and 3) was achieved when the reaction was carried out in ethanol or *n*-PrOH. However, neither reducing the pH of the solution nor changing HCl to CH_3_COOH improved the reaction yield (Table 3, entries 4 and 5).

**Table 3:** Optimization of Mannich cyclization.^a^

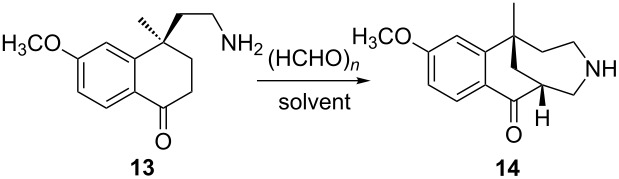				

entry	solvent	acid (pH^b^)	temperature	yield

1	MeOH	HCl (5–6)	reflux	25.6%
2	EtOH	HCl (5–6)	reflux	45.5%
3	*n*-PrOH	HCl (5–6)	reflux	46.2%
4	EtOH	HCl (1–2)	reflux	42.1%
5	EtOH	CH_3_COOH	reflux	complex mixture

^a^The reaction was performed with **13** and paraformaldehyde (6 equiv) for 6 h. ^b^pH of the reaction solution.

The reductive methylation of **14** under Eschweiler–Clarke conditions (HCOOH/formalin/reflux) furnished **15** in quantitative yield. The latter was reduced by NaBH_4_ in methanol at room temperature, and then dehydration and hydrogenation with H_2_/Pd/C in acetic acid gave compound **8** in 83% yield. Eventually, target compound **1** was obtained from **8** via demethylation with 48% aqueous HBr solution.

## Conclusion

In summary, we developed a new and efficient synthesis of eptazocine hydrobromide involving enantioselective phase-transfer catalyzed alkylation of 1-methyl-7-methoxy-2-tetralone and a Mannich reaction to construct the tricyclic compound in eight linear steps. The optimization of reaction conditions were carried out to get a practical route delivering the product in excellent yield and purity (>99%).

## Experimental

All solvents and reagents were from commercial sources and used without further purification. Catalyst **3** was prepared according to the literature [14]. Compound **2** was prepared according to the literature [19]. Melting points were determined on a Büchi melting point M-565 apparatus and are uncorrected. ^1^H NMR spectra were recorded using a Bruker 400 MHz spectrometer with TMS as an internal standard. Mass spectra were recorded with a Q-TOF mass spectrometer using electrospray positive ionization (ESI^+^). Enantiomeric ratios were determined by HPLC using a chiral column (Chiralpak AY-H) with hexane/isopropyl alcohol 50:50 as eluents, detected at 210 nm. Specific rotations were determined on a Rudolph Research Analytical automatic polarimeter IV. All reactions were monitored by TLC, which were carried out on silica gel GF254 plates. Column chromatography was carried out on silica gel (HF254) purchased from Qingdao Ocean Chemical Company of China.

### 

#### Preparation of (*R*)-1-methyl-1-cyanomethyl-7-methoxy-2-tetralone (**4**)

To a solution of **2** (60 g, 0.315 mol) and catalyst **3** (16.8 g, 0.031 mol) in chlorobenzene (4 L) was added 30% aq NaOH (450 mL) at 0 °C under N_2_ atmosphere. After this mixture was stirred for 20 min, a solution of chloroacetonitrile (47.6 g, 0.63 mol) in chlorobenzene (500 mL) was added dropwise over 3 h at 0–5 °C. After the reaction was complete, the aqueous layer was separated and extracted with chlorobenzene (400 mL). The combined organic layers were washed with 1 M aqueous HCl solution (2 L) and water (2 L), and then the solvent was removed under reduced pressure to afford the product as a white solid (57.0 g, 78.8% yield), chiral purity (HPLC): **4**/**4’** 80:20. The mixture was crystallized from ethyl acetate (120 mL) to afford **4** as a white solid (34.7 g, 48.0%). Chiral purity (HPLC): 99.92%. Mp 104–105 °C; [α]_D_^20^ +102 (*c* 1, CHCl_3_); ^1^H NMR (400 MHz, CDCl_3_) δ 7.26 (d, *J* = 8.0 Hz, 1H), 6.88 (s, 1H), 6.75–6.73 (d, *J* = 8.0 Hz, 1H), 3.82 (s, 3H), 3.10–3.07 (m, 2H), 3.03–2.99 (d, 1H), 2.83–2.77 (m, 2H), 2.70–2.65 (m, 1H), 1.51 (s, 3H); ^13^C NMR (100 MHz, CDCl_3_) δ 209.98, 157.95, 139.01, 128.70, 126.73, 116.67, 111.81, 111.06, 54.37, 48.86, 36.18, 26.52, 26.06, 25.82; MS (ES^+^) *m*/*z*: 252.13 [M + Na]^+^.

#### Preparation of (*S*)-1-methyl-1-cyanomethyl-7-methoxy-1,4-dihydronaphthalene (**11**)

To a solution of **4** (20 g, 0.087 mol) in MeOH (200 mL) was added NaBH_4_ (1.98 g, 0.053 mol) in portions at 0 °C. The mixture was stirred at 0 °C for 20 min and then acidified with acetic acid (20 mL) to pH 6. After evaporation of the solvent under reduced pressure, the residue was taken up in dichloromethane (200 mL) and washed with 10% aq Na_2_CO_3_ solution (200 mL). The aqueous layer was extracted with dichloromethane (200 mL). The combined organic layers were dried, filtered and concentrated to yield the hydroxy intermediate (20 g, 99%). To a solution of this intermediate in pyridine (150 mL) was added POCl_3_ (20 g, 0.13 mol). After refluxing for 40 min, the mixture was concentrated. Ice water (200 mL) was added to the residue, and the mixture was acidified with 6 mol/L hydrochloric acid solution to pH 1. Then, the aqueous layer was extracted with dichloromethane (200 mL × 3). The combined organic layers were dried, filtered through a pad of silica gel and evaporated under reduced pressure to give **11** as a white solid (15.8 g, 85.0%). [α]_D_^20^ +103 (*c* 1, CHCl_3_); ^1^H NMR (400 MHz, CDCl_3_) δ 7.10–7.08 (d, *J* = 8.0 Hz, 1H), 6.88 (s, 1H), 6.81–6.78 (dd, *J* = 8.0 Hz, 1H), 6.08–6.04 (m, 1H), 5.73–5.70 (d, 1H) 3.81 (s, 3H), 3.47–3.40 (m, 2H), 2.60 (d, 2H), 1.53 (s, 3H); MS (ES^+^) *m*/*z*: 214.05 [M + H]^+^.

#### Preparation of (*S*)-4-cyanomethyl-6-methoxy-4-methyl-1,4-dihydronaphthalen-1-one (**12**)

To a solution of **11** (30 g, 0.14 mol) in THF (300 mL) and water (3 mL) was added in portions DDQ (63.8 g, 0.28 mol) with stirring at 0 °C. The mixture was stirred at room temperature for 2 h and evaporated under reduced pressure to dryness. The residue was taken up in dichloromethane (300 mL), stirred for 20 min and then filtered through a pad of diatomite. The filtrate was washed with 5% aq NaOH (150 mL × 2) and water (150 mL), dried, filtered and then concentrated to dryness. The residue was purified through recrystallization with 90 mL ethyl alcohol to afford **12** as a white solid (22.4 g, 70.1%). Mp 101–102 °C; [α]_D_^20^ +111 (*c* 1, CHCl_3_); ^1^H NMR (400 MHz, CDCl_3_) δ 8.12–8.09 (d, *J* = 12.0 Hz, 1H), 6.93–6.87 (m, 2H), 6.83–6.80 (d, *J* = 12.0 Hz, 1H), 6.42–6.40 (d, *J* = 8.0 Hz, 1H), 2.76–2.63 (m, 2H), 1.56 (s, 3H); ^13^C NMR (100 MHz, CDCl_3_) δ 181.99, 162.45, 149.57, 146.13, 128.86, 128.22, 123.42, 115.47, 112.79, 110.00, 54.61, 37.81, 30.28, 26.35; MS (ES^+^) *m*/*z*: 228.05 [M + H]^+^.

#### Preparation of (*R*)-4-(2-aminoethyl)-6-methoxy-4-methyl-1,2,3,4-tetrahydronaphthalen-1-one (**13**)

A solution of **12** (6 g, 0.0264 mol) in MeOH (30 mL) and 7 mol/L methanol solution of ammonia (60 mL) was subjected to hydrogenation in the presence of Raney-Ni (1.8 g, 30%) under hydrogen (pressure 0.7 MPa) at room temperature for 16 h. After the hydrogen absorption ceased, the catalyst was removed by filtration. The filtrate was concentrated to dryness, and then the residue was taken up in ethyl acetate (100 mL), washed with water (50 mL) and saturated saline solution (50 mL). The organic layers were dried, filtered and concentrated to yield a crude oil. The crude oil was purified through salt formation with tartaric acid (3.96 g, 0.026 mol) in ethyl alcohol (60 mL) and basification to afford **13** as colorless oil (4.49 g, 73.0%). [α]_D_^22^ −27.7 (*c* 1, EtOH); ^1^H NMR (400 MHz, CDCl_3_) δ 8.05–8.02 (d, *J* = 12.0 Hz, 1H), 6.83–6.81 (m, 2H), 3.87 (s, 3H), 2.74–2.62 (m, 4H), 2.08–2.07 (m, 1H), 1.96–1.90 (m, 3H), 1.48 (s, 2H), 1.38 (s, 3H); ^13^C NMR (100 MHz, CDCl_3_) δ 196.80, 163.80, 153.46, 130.11, 125.16, 111.74, 111.16, 55.35, 45.01, 37.90, 36.40, 34.36, 34.25, 27.44; MS (ES^+^) *m*/*z*: 234.24 [M + H]^+^.

#### Preparation of (1*S*,6*R*)-1-methyl-10-methoxy-2,3,4,5-tetrahydro-1*H*-1,6-methanobenzo[*e*]azonin-7(6*H*)-one (**14**)

To a solution of **13** (4.5 g, 0.019 mol) in absolute ethyl alcohol (90 mL) acidified with 28% ethanol solution of HCl to pH 5–6 was added paraformaldehyde (3.1 g, 0.103 mol). The mixture was stirred at reflux for 6 h and evaporated under reduced pressure to dryness. Water (45 mL) was added to the residue, and the mixture was alkalified with 50% NaOH (aq) to pH 11, and then extracted with ethyl acetate (45 mL × 5). The combined organic layers were washed with water (100 mL × 2), dried, filtered and evaporated under reduced pressure to dryness. The residue was purified via flash chromatography with dichloromethane/methanol (20:1) to give **14** as colorless oil (2.15 g, 45.5%). ^1^H NMR (400 MHz, CDCl_3_) δ 8.07–8.05 (d, *J* = 8.0 Hz, 1H), 6.88–6.86 (m, 2H), 3.87 (s, 3H), 3.30–3.26 (m, 2H), 2.89–2.84 (m, 2H), 2.23–2.09 (m, 4H), 1.90–1.79 (m, 2H), 1.46 (s, 3H); MS (ES^+^) *m*/*z*: 246.20 [M + H]^+^.

#### Preparation of (1*S*,6*R*)-1,4-dimethyl-10-methoxy-2,3,4,5-tetrahydro-1*H*-1,6-methanobenzo[*e*]azonin-7(6*H*)-one (**15**)

To a solution of **14** (4.0 g, 0.016 mol) and formic acid (7.5 g, 0.16 mol) in water (28 mL) was added paraformaldehyde (4.9 g, 0.16 mol). The mixture was stirred at reflux for 2 h and then alkalified with 30% NaOH (aq) to pH 11. The aqueous layer was extracted with ethyl acetate (30 mL × 3), and the combined organic layers were washed with water (30 mL × 2), dried, filtered and evaporated under reduced pressure to afford **15** as colorless oil (4.23 g, 100%). [α]_D_^20^ +5.2 (*c* 1.1, EtOH); [4] [α]_D_^20^ +5.1 (*c* 1.15, EtOH); ^1^H NMR (400 MHz, CDCl_3_) δ 8.05–8.03 (d, *J* = 8.0 Hz, 1H), 6.87–6.84 (m, 2H), 3.87 (s, 3H), 3.15 (t, 1H), 2.85–2.80 (m, 2H), 2.41–2.33 (m, 4H), 2.19–2.16 (m, 1H), 2.07–2.03 (m, 2H), 1.68–1.66 (m, 1H) 1.44 (s, 3H); MS (ES^+^) *m*/*z*: 260.20 [M + H]^+^.

#### Preparation of (1*S*,6*S*)-1,4-dimethyl-10-methoxy-2,3,4,5,6,7-hexahydro-1*H*-1,6-methanobenzo[*e*]azonine (**8**)

To a solution of **15** (3.0 g, 11.56 mmol) in MeOH (40 mL) was added NaBH_4_ (0.48 g, 12.70 mmol) with stirring at 0 °C. The mixture was stirred at room temperature for 2 h and then acidified with 2 M hydrochloric acid solution to pH 6. The solvent was evaporated under reduced pressure. The residue was taken up in water (30 mL), alkalified with 30% NaOH (aq) to pH 11. The aqueous layer was extracted with ethyl acetate (30 mL × 3). The combined organic layers were dried, filtered and evaporated under reduced pressure to yield white solid (3.0 g). To a solution of the above solid and methanesulfonic acid (0.96 g, 11.56 mmol) in acetic acid (20 mL) was added 10% palladium on carbon (0.90 g, 30%). The mixture was subjected to hydrogenation at room temperature in atmosphere pressure for 12 h. After the catalyst was removed by filtration, the filtrate was concentrated under reduced pressure. Water (30 mL) was added to the residue, and the mixture was alkalified with 30% NaOH (aq) to pH 12. The aqueous layer was extracted with dichloromethane (30 mL × 3). The combined organic layers were dried, filtered and evaporated under reduced pressure to yield **8** as colorless oil (2.36 g, 83.4%). [α]_D_^20^ −16.2 (*c* 1, EtOH); [4] [α]_D_^23^ −16.0 (*c* 1, EtOH); ^1^H NMR (400 MHz, CDCl_3_) δ 6.95–6.93 (d, *J* = 8.0 Hz, 1H), 6.75–6.74 (d, 1H), 6.66–6.63 (m, 1H), 3.74 (s, 3H), 2,98 (t, 1H), 2.78–2.74 (m, 1H), 2.56–2.55 (m, 1H), 2.35 (m, 1H), 2.31 (m, 1H), 2.21–2.17 (m, 4H), 1.80–1.60 (m, 5H), 1.20 (s, 3H); MS (ES^+^) *m*/*z*: 246.17 [M + H]^+^.

#### Preparation of (1*S*,6*S*)-1,4-dimethyl-2,3,4,5,6,7-hexahydro-1*H*-1,6-methanobenzo[*e*]azonine-10-ol hydrobromide (**1**)

A solution of **8** (2.0 g, 8.14 mmol) in 48% hydrobromic acid (16 mL) was refluxed for 2 h under N_2_ atmosphere. After the reaction was completed, the solvent was evaporated under reduced pressure. The residue was recrystallized from ethanol (12 mL) to afford **1** as white crystals (2.0 g, 80%), HPLC purity >99%. Mp 260–261 °C; [α]_D_^22^ −15.3 (*c* 4.87, water); [4] mp 259.3 °C; [α]_D_^25^ −15.4 (*c* 4.87, water); ^1^H NMR (400 MHz, CH_3_OD) δ 9.16 (s, 1H), 6.94–6.91 (d, *J* = 12.0 Hz, 1H), 6.71 (d, 1H), 6.63–6.60 (m, 1H), 3.69–3.61 (t, 1H), 3.32–3.27 (m, 2H), 2,76 (m, 4H), 2.57–2.55 (m, 1H), 2.41–2.37 (m, 2H), 1.90–1.80 (m, 3H), 1.24 (s, 3H); MS (ES^+^) m/*z*: 232.18 [M + H]^+^.

## Supporting Information

File 1 ^1^H NMR and MS spectra of **3**, **4**, **8**–**15**, ^13^C NMR spectra of **4**, **12**, **13**, chiral HPLC chromatograms of **4**, ^1^H NMR, MS and HPLC chromatograms of **1**.
