# Supplementary material for: A novel and practical asymmetric synthesis of eptazocine hydrobromide
Source: Beilstein J Org Chem. 2018 Sep 6;14:2340–7. doi: 10.3762/bjoc.14.209 (PMC6142761; doi:10.3762/bjoc.14.209)
Supplement: File 1 — 1H NMR and MS spectra of 3, 4, 8–15, 13C NMR spectra of 4, 12, 13, chiral HPLC chromatograms of 4, 1H NMR, MS and HPLC chromatograms of 1. [file Beilstein_J_Org_Chem-14-2340-s001.pdf]

Supporting Information

for

A novel and practical asymmetric synthesis of eptazocine hydrobromide

Ruipeng Li, Zhenren Liu, Liang Chen, Jing Pan, Kuaile Lin and Weicheng Zhou*

Address: State Key Lab of New Drug & Pharmaceutical Process, Shanghai Key Lab of Anti-Infectives, Shanghai Institute of Pharmaceutical Industry, China State Institute of Pharmaceutical Industry, No. 285, Gebaini Rd., Shanghai 201203, P. R. of China

Email: Weicheng Zhou* - zhouweicheng58@163.com

* Corresponding author

^{1}H NMR and MS spectra of 3, 4, 8–15, ^{13}C NMR spectra of 4, 12, 13, chiral HPLC chromatograms of 4, ^{1}H NMR, MS and HPLC chromatograms of 1

Table of Contents

^{1}H NMR and MS data of **3**, **4**, **8–15** S2–S14

^{13}C NMR spectra of **4**, **12**, **13** S5, S10–S11

Chiral HPLC chromatograms of **4** S15

^{1}H NMR, MS and HPLC chromatograms of **1** S16–S17

^{1}H NMR and MS data of catalyst 3:

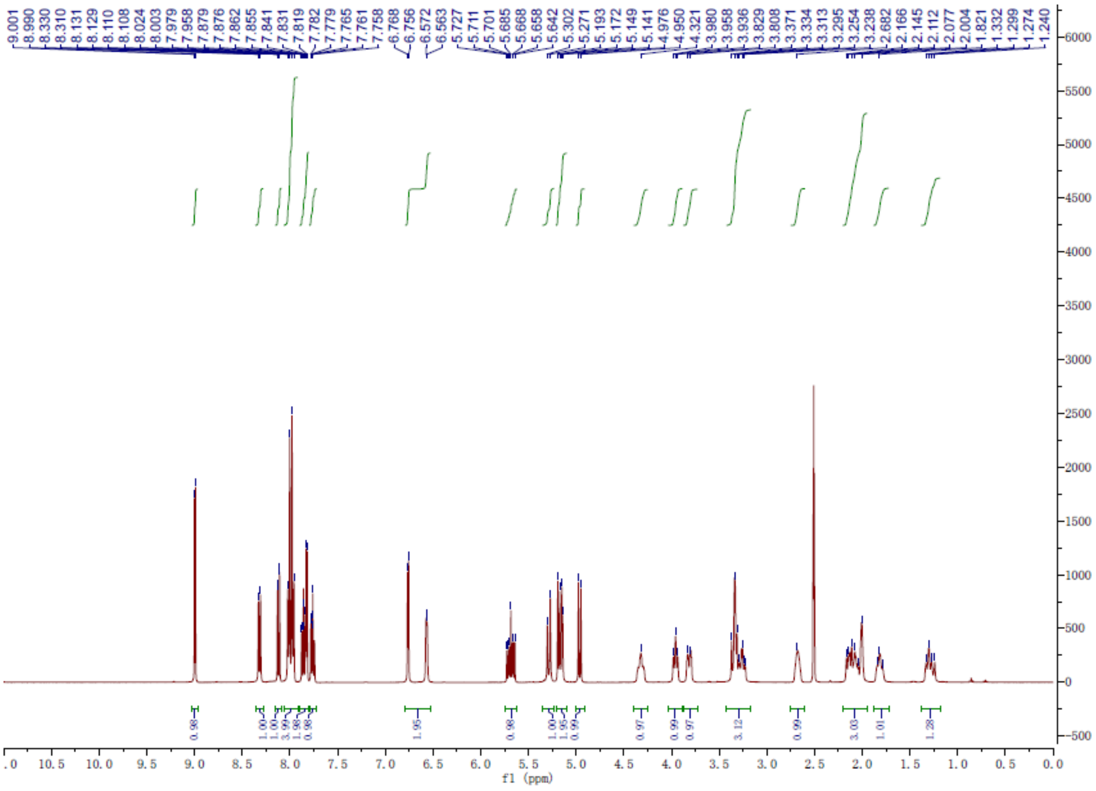

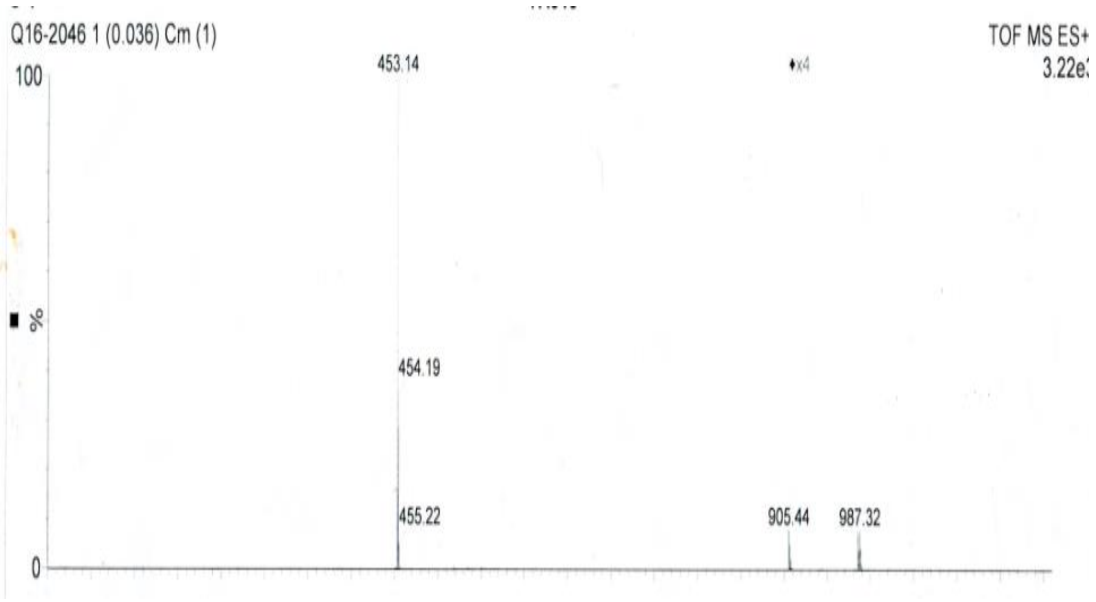

^{1}H NMR, ^{13}C NMR and MS data of 4:

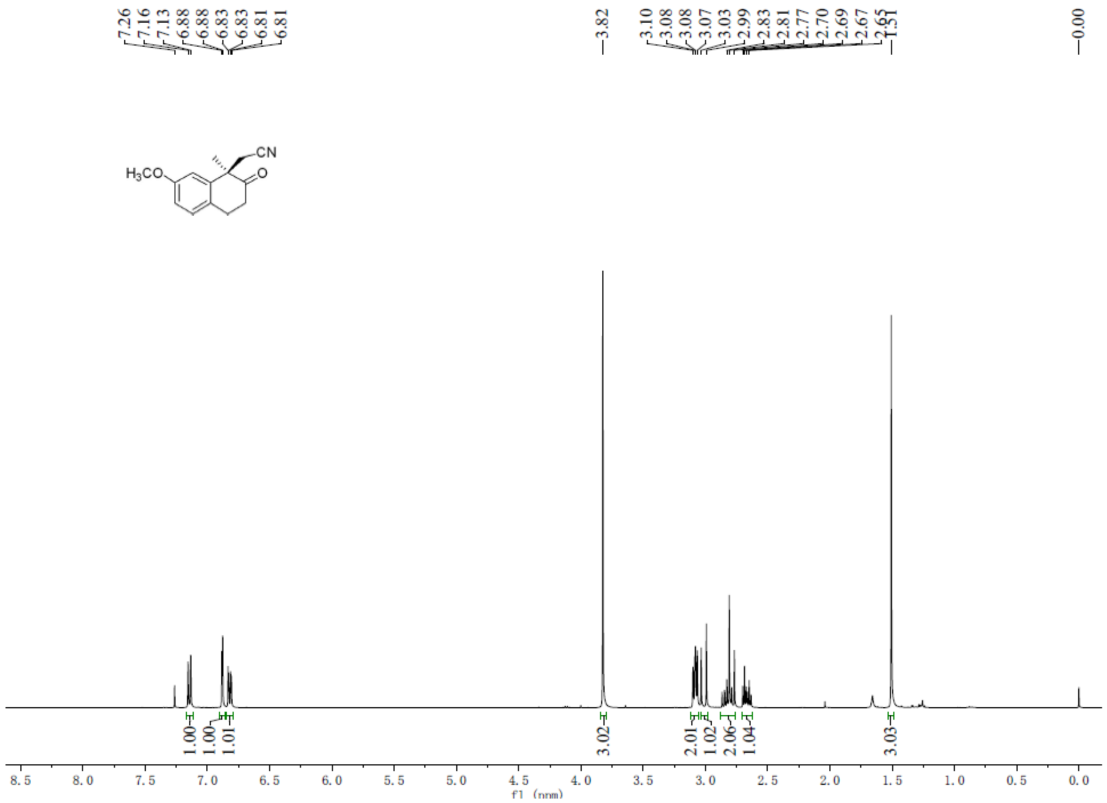

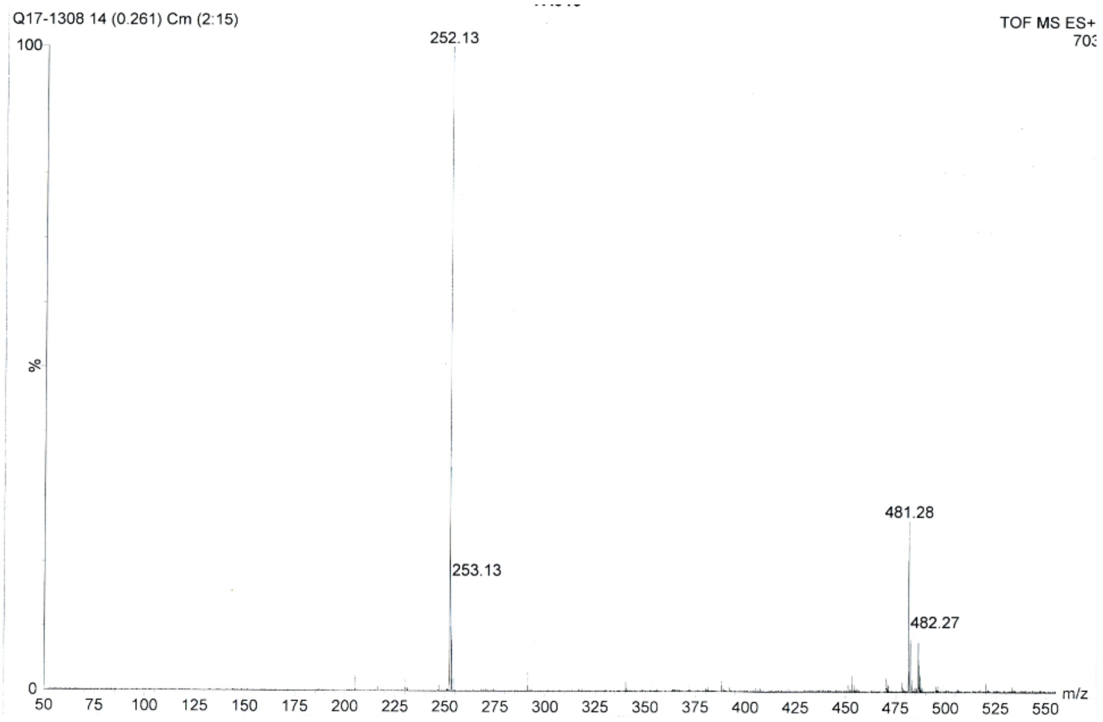

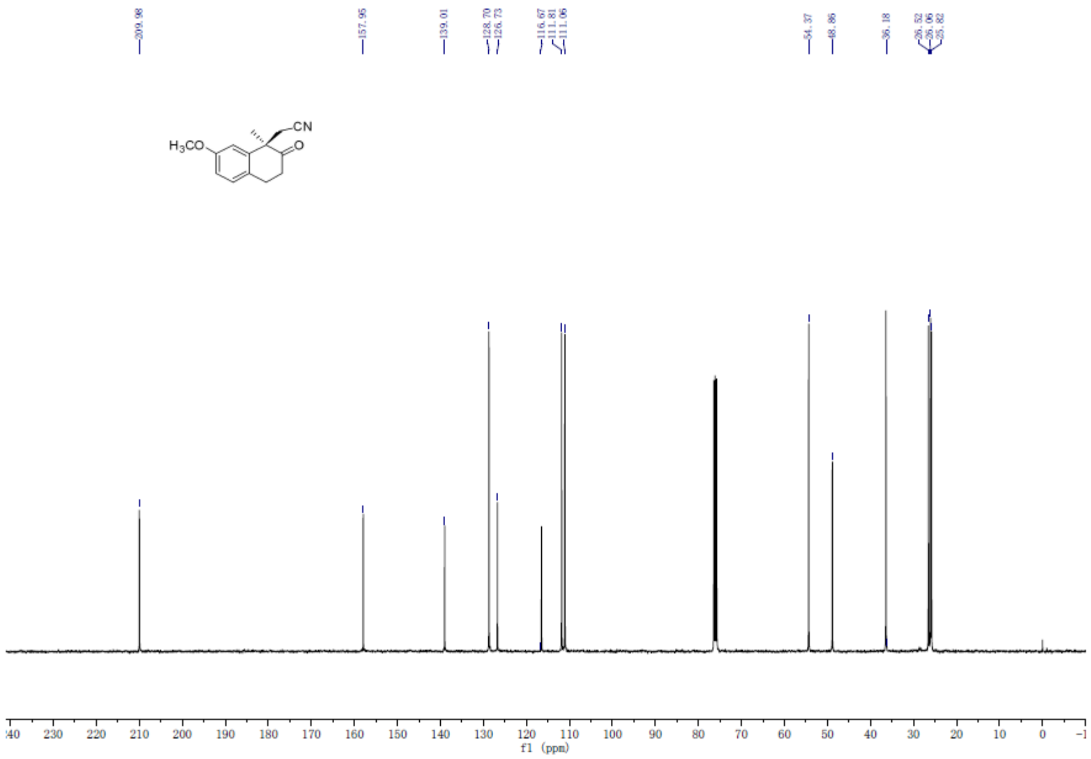

^{1}H NMR and MS data of 9:

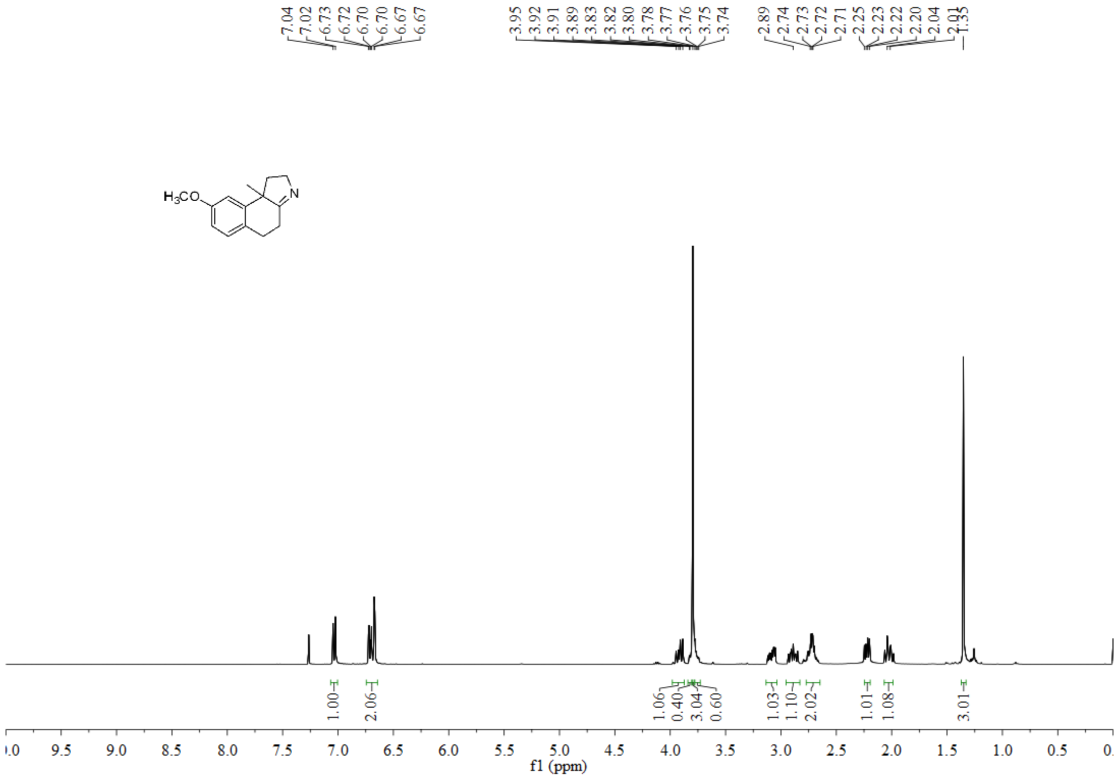

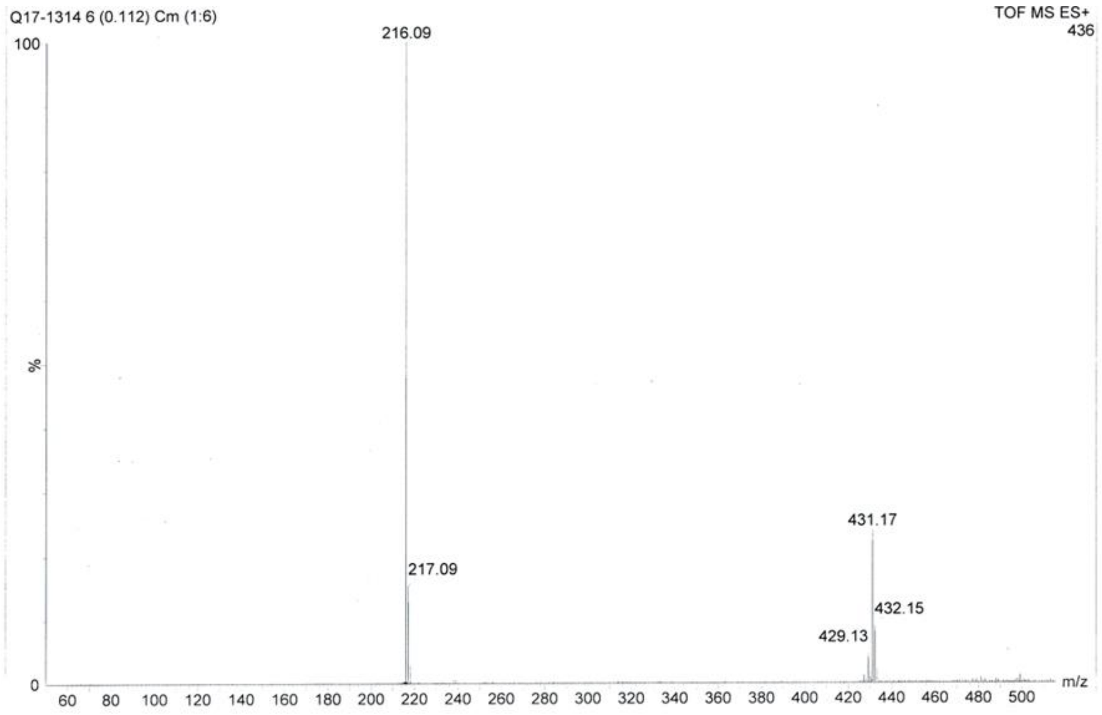

^{1}H NMR and MS data of 10:

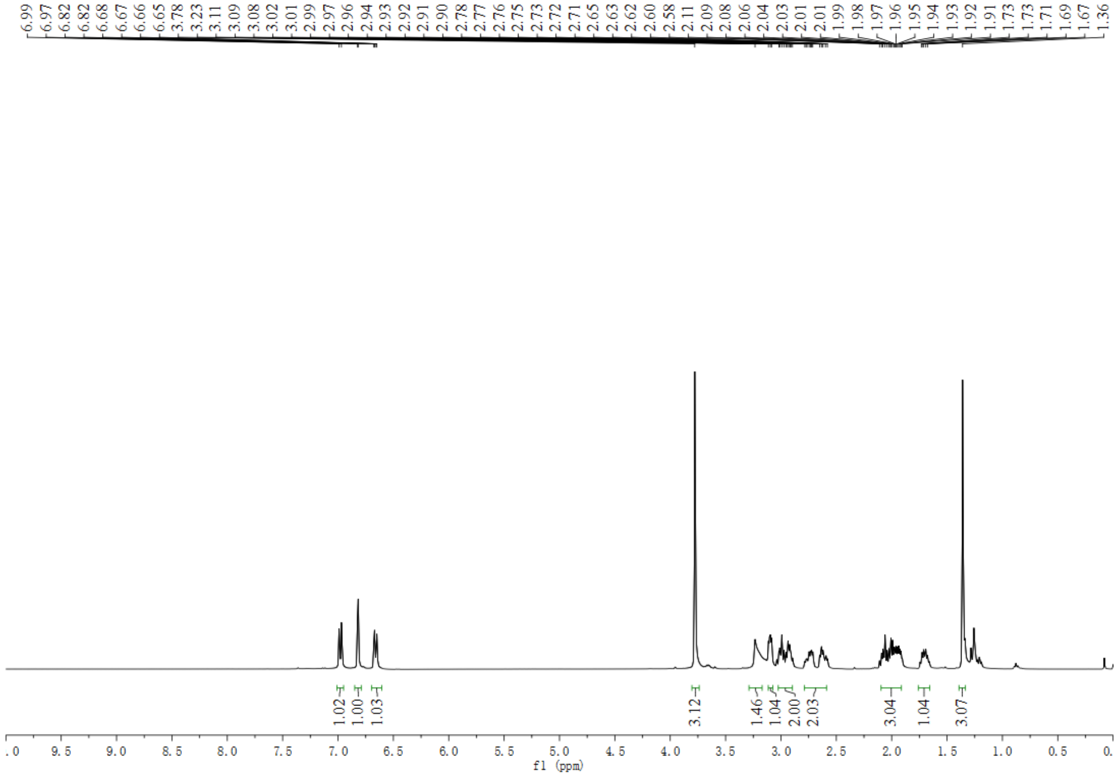

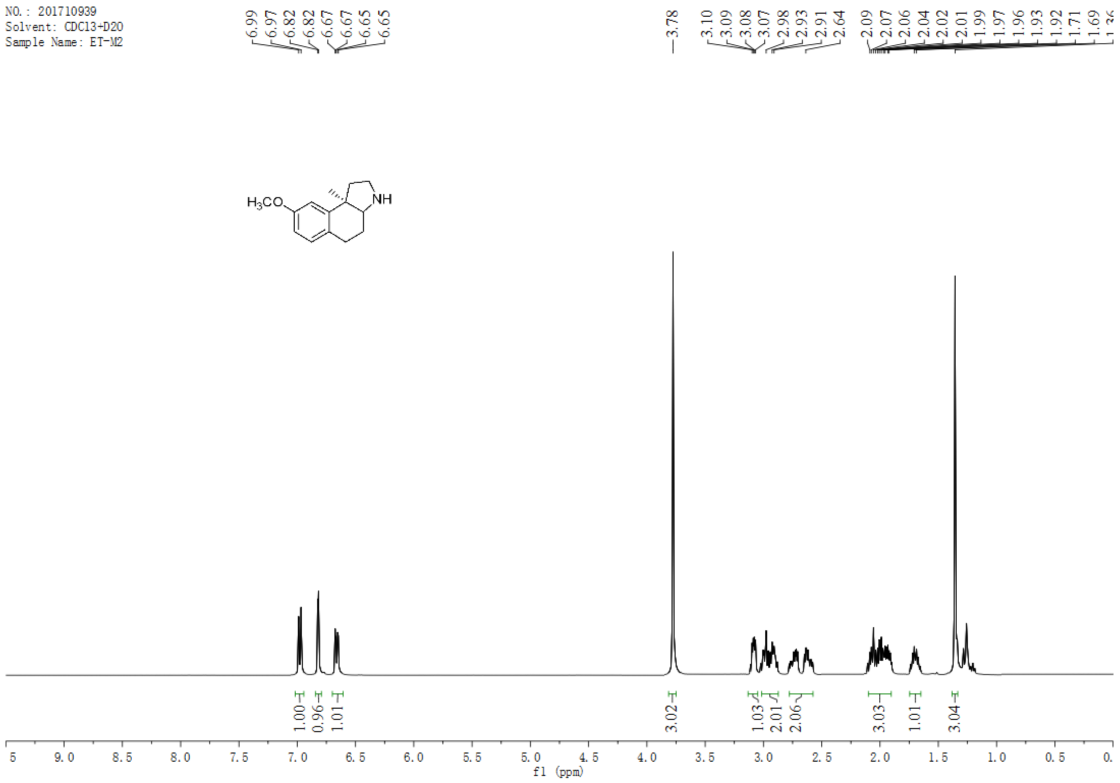
NO.: 201710939
Solvent: CDCl3+D2O
Sample Name: ET-M2
H3CO
NH
f1 (ppm)

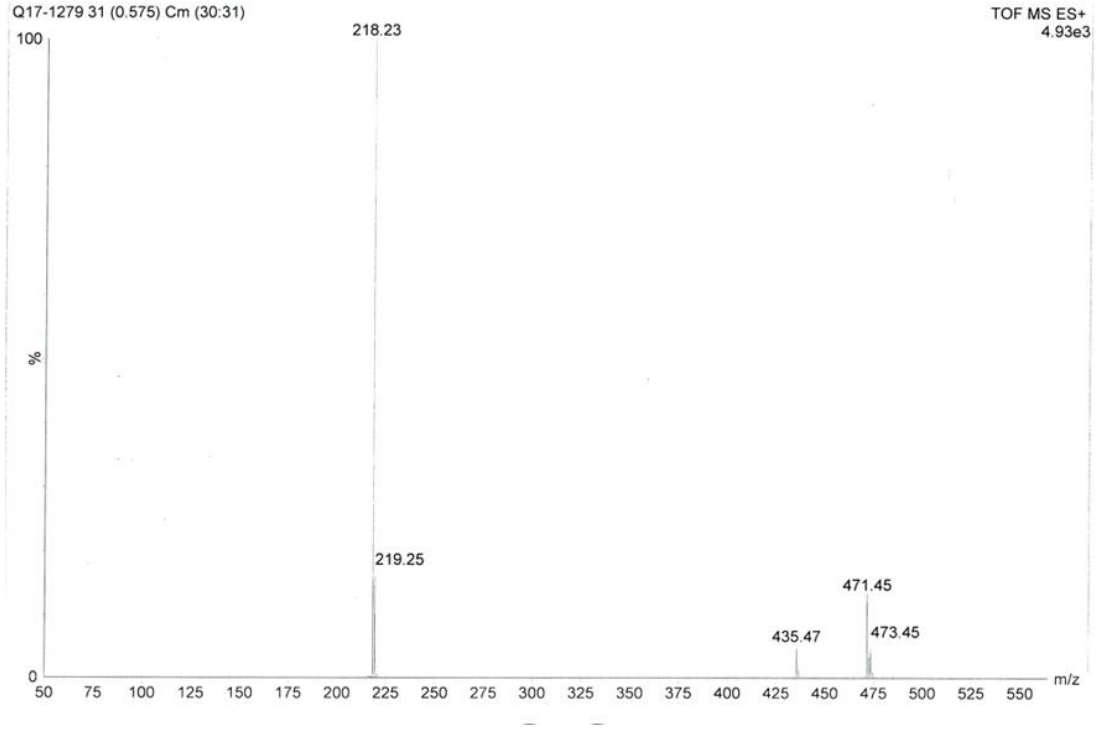
Q17-1279 31 (0.575) Cm (30:31)
TOF MS ES+
4.93e3
218.23
219.25
471.45
435.47
473.45
%
m/z

^{1}H NMR and MS data of 11:

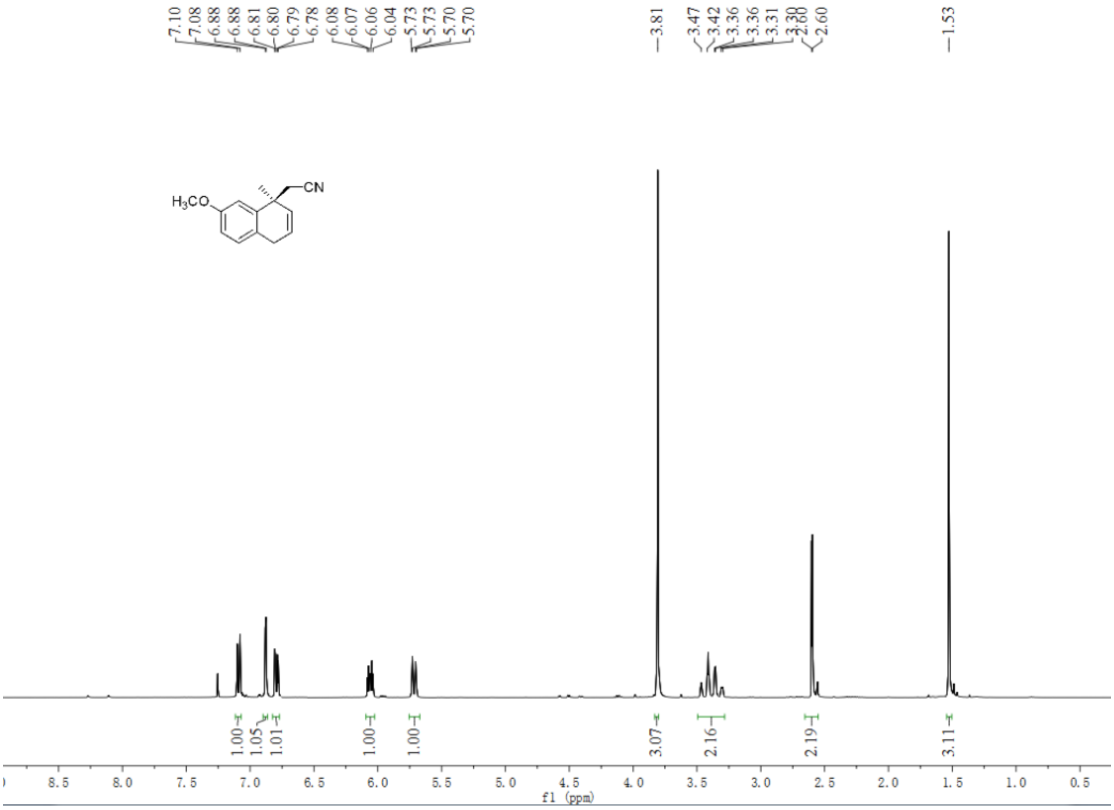

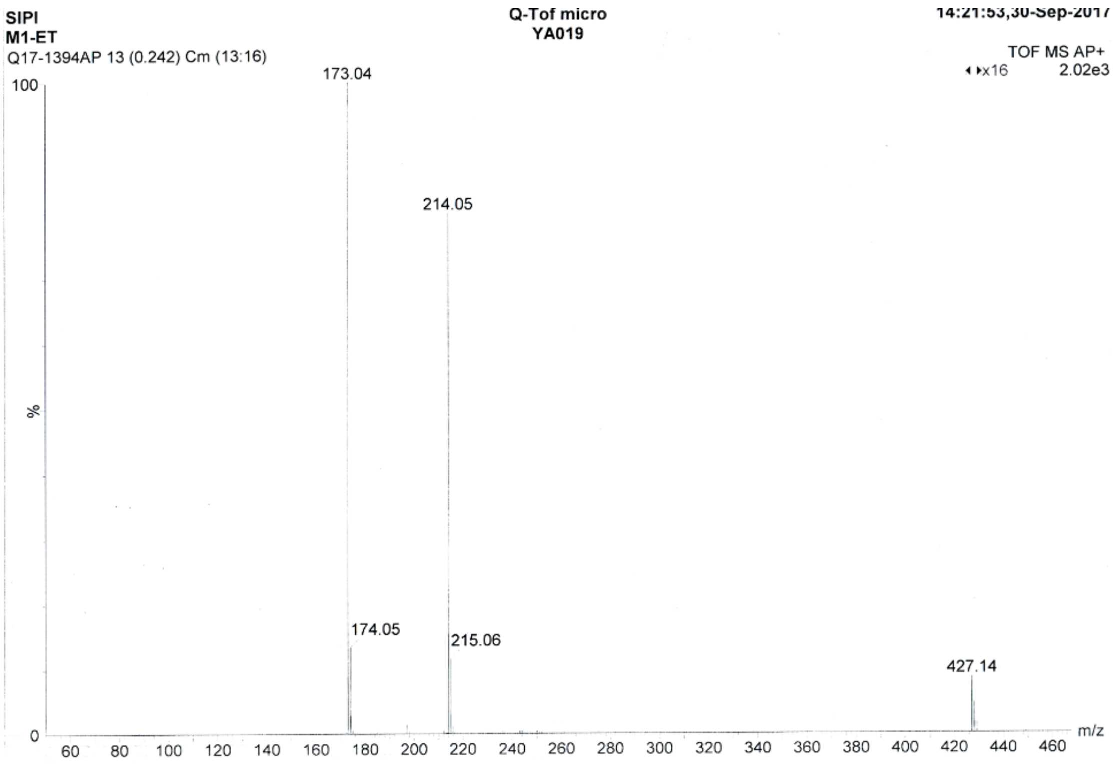

^{1}H NMR, ^{13}C NMR and MS data of 12:

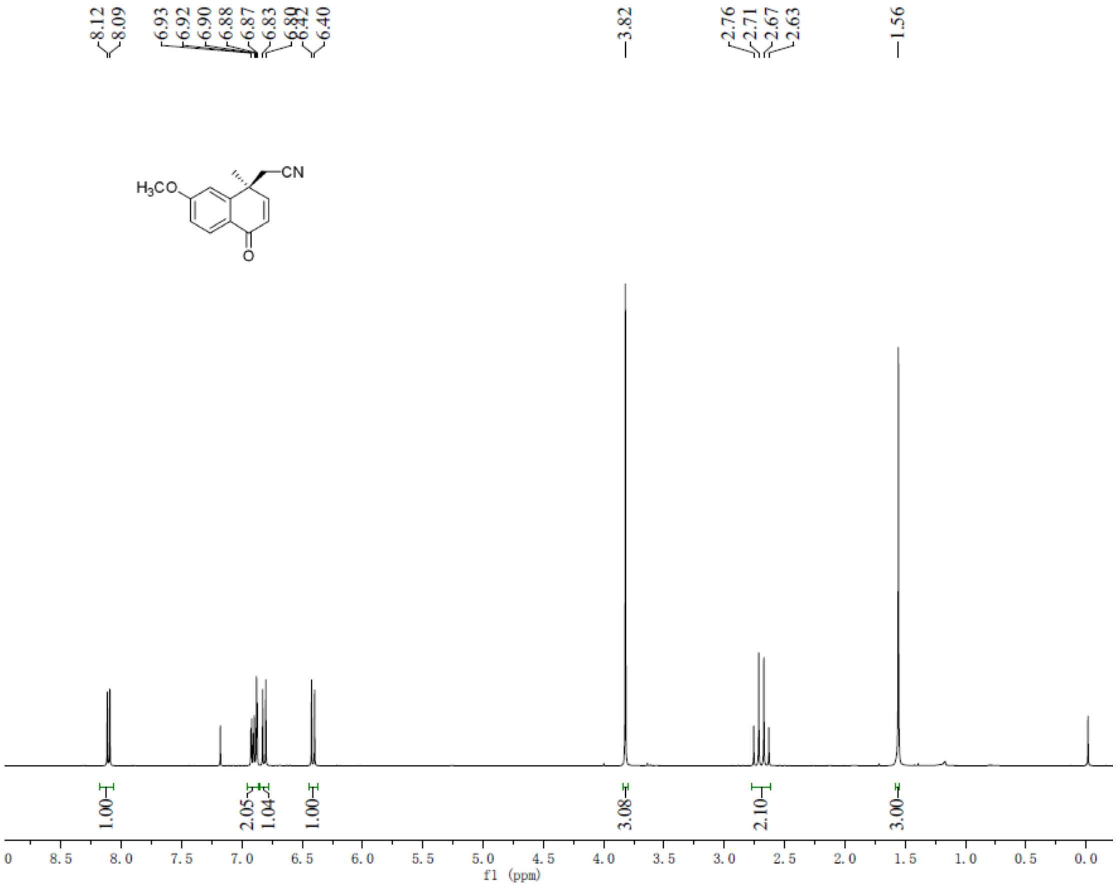

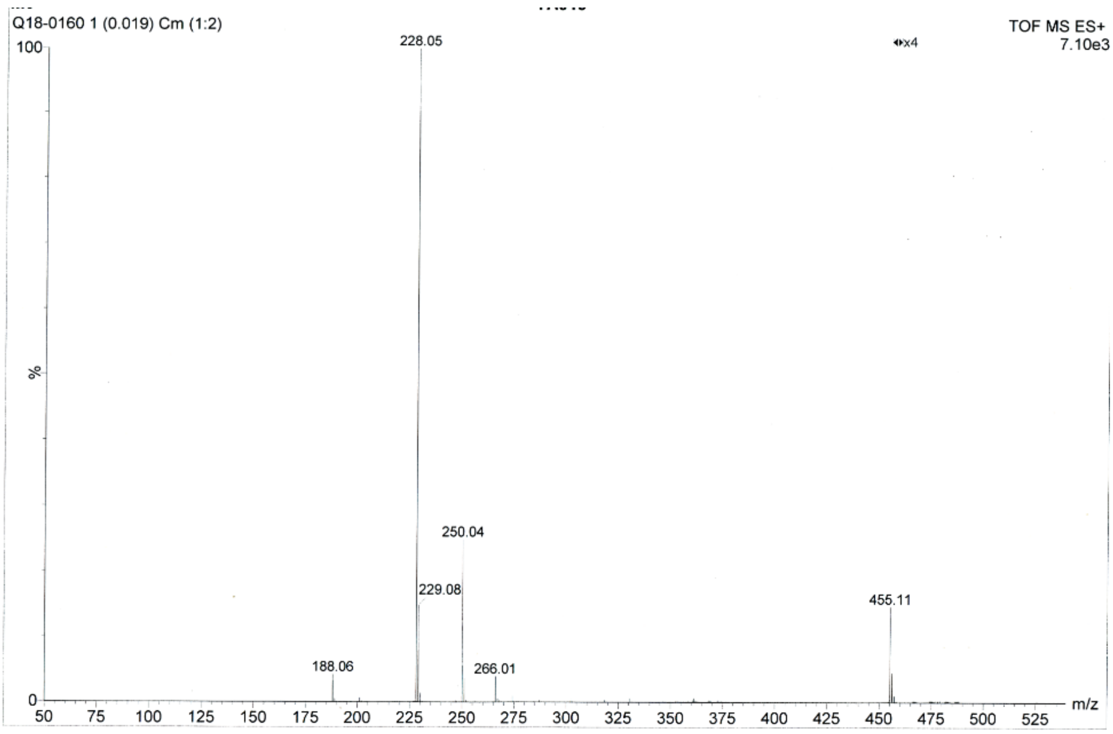

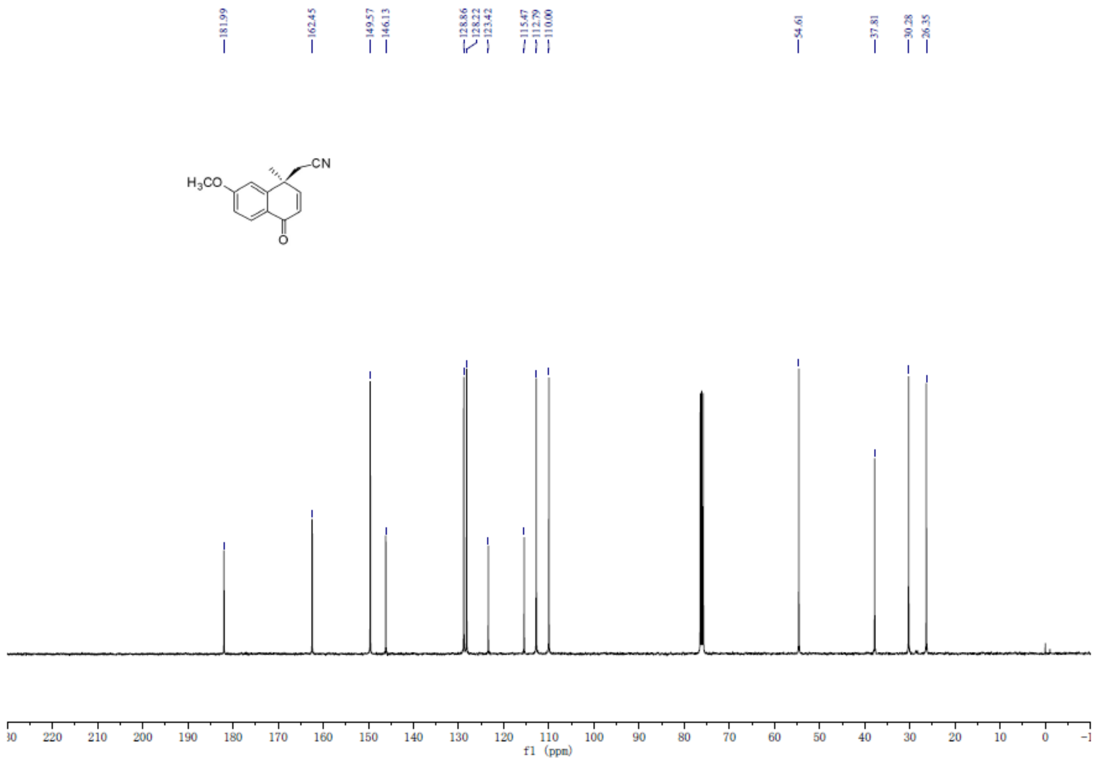

^{1}H NMR, ^{13}C NMR and MS data of 13:

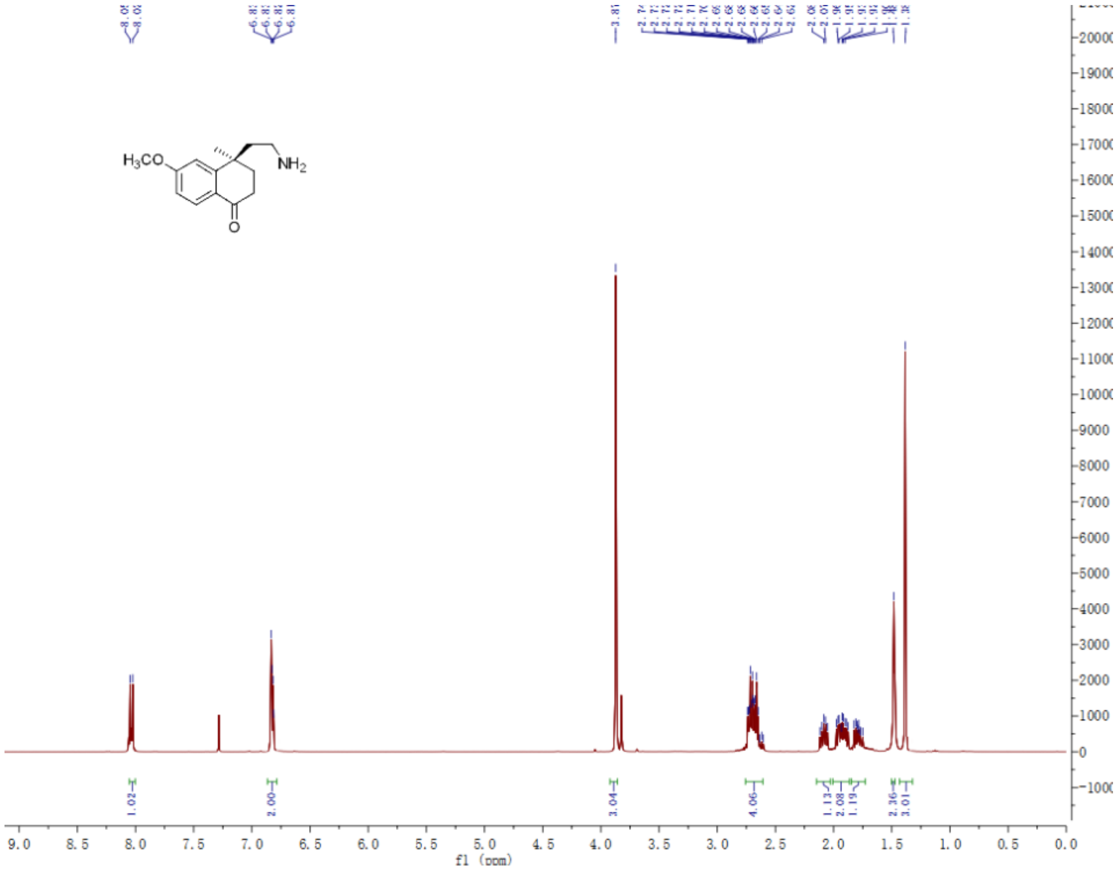

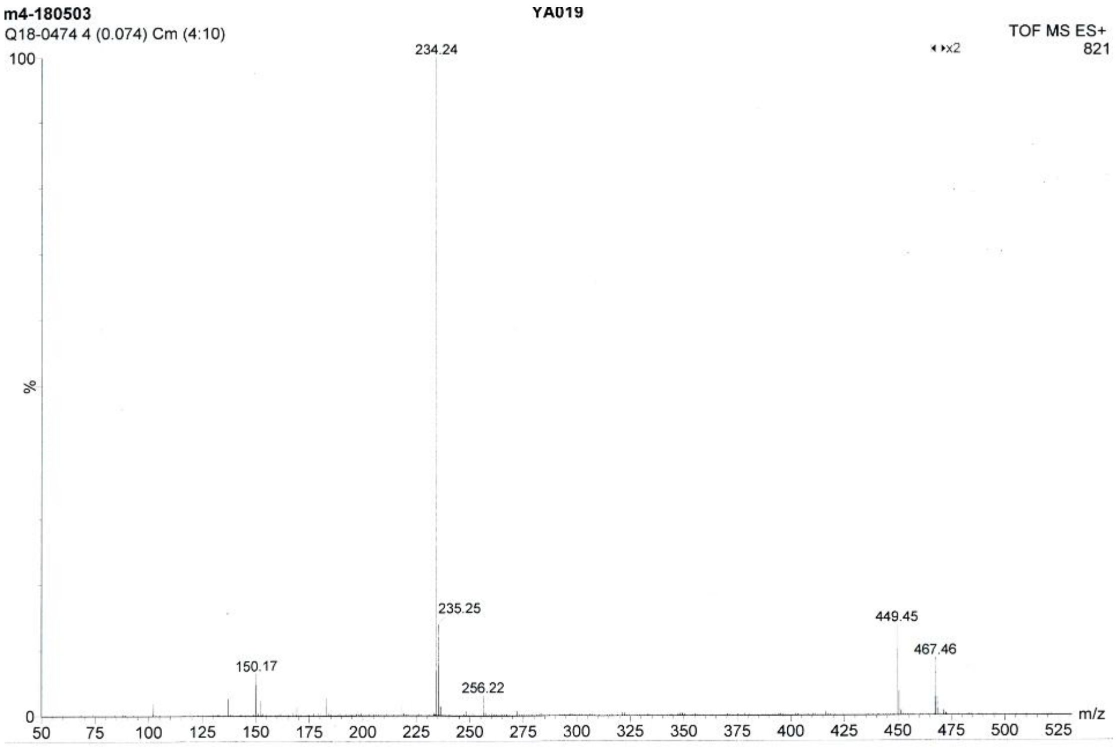

m4-180503
YA019
Q18-0474 4 (0.074) Cm (4:10)
TOF MS ES+
821
x2
100
%
0
234.24
235.25
150.17
256.22
449.45
467.46
m/z
50
75
100
125
150
175
200
225
250
275
300
325
350
375
400
425
450
475
500
525

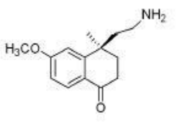

NH2
H3CO
O

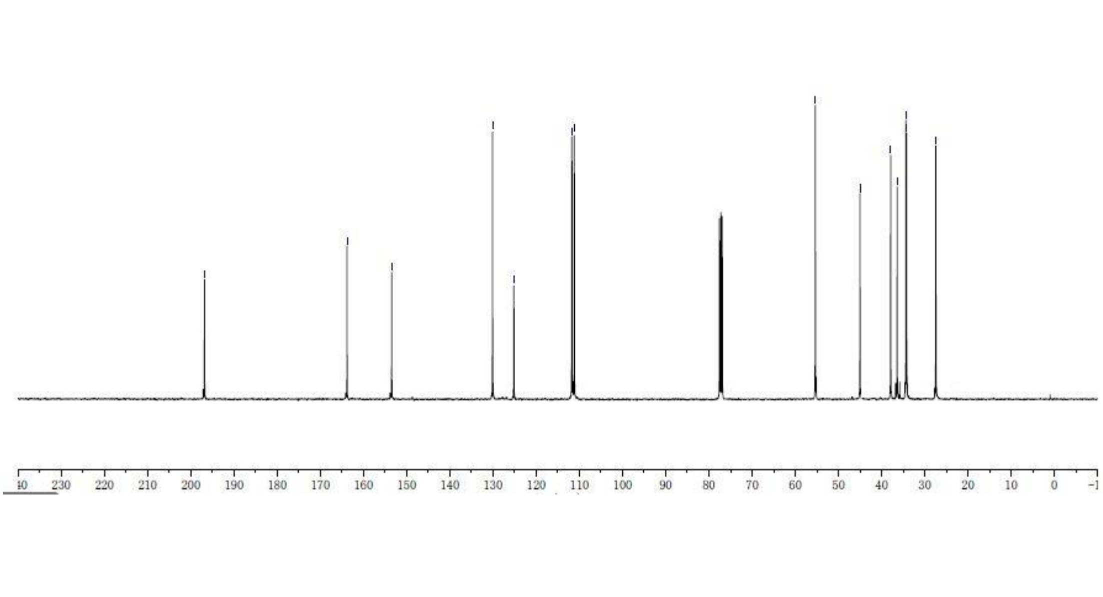

196.80
163.80
153.46
130.11
125.16
111.74
111.16
55.35
45.01
37.90
36.40
34.36
34.25
27.44

^{1}H NMR and MS data of 14:

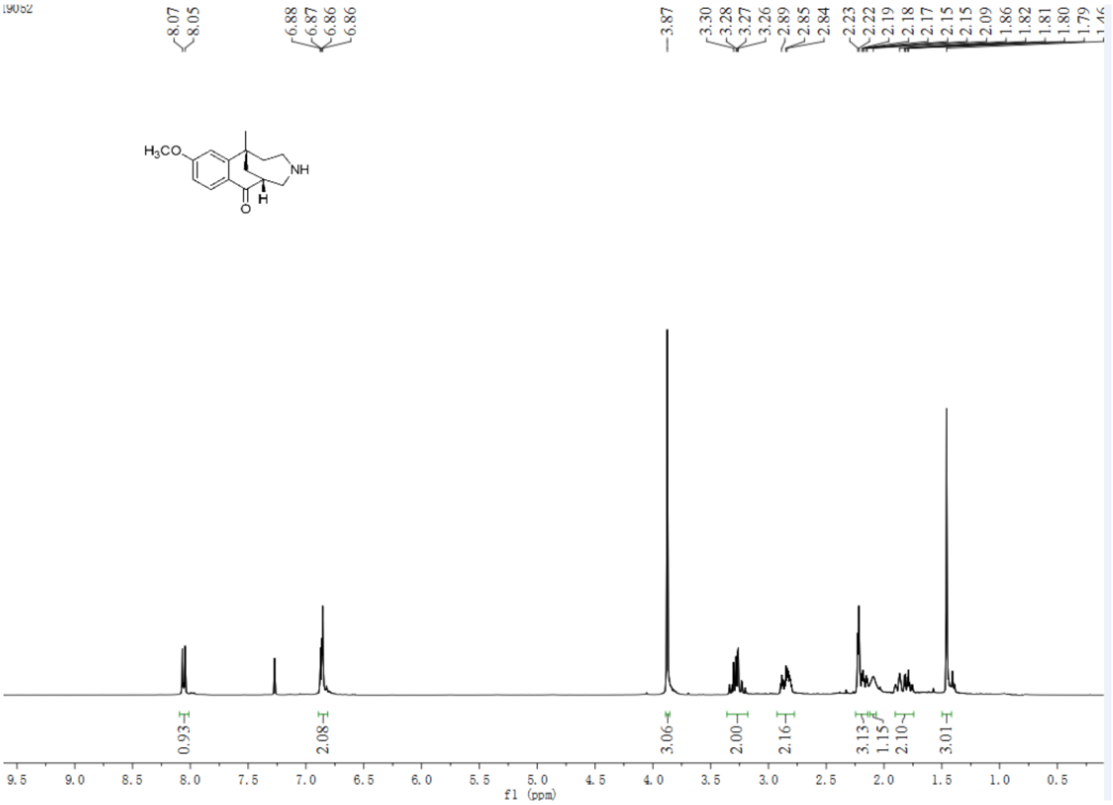

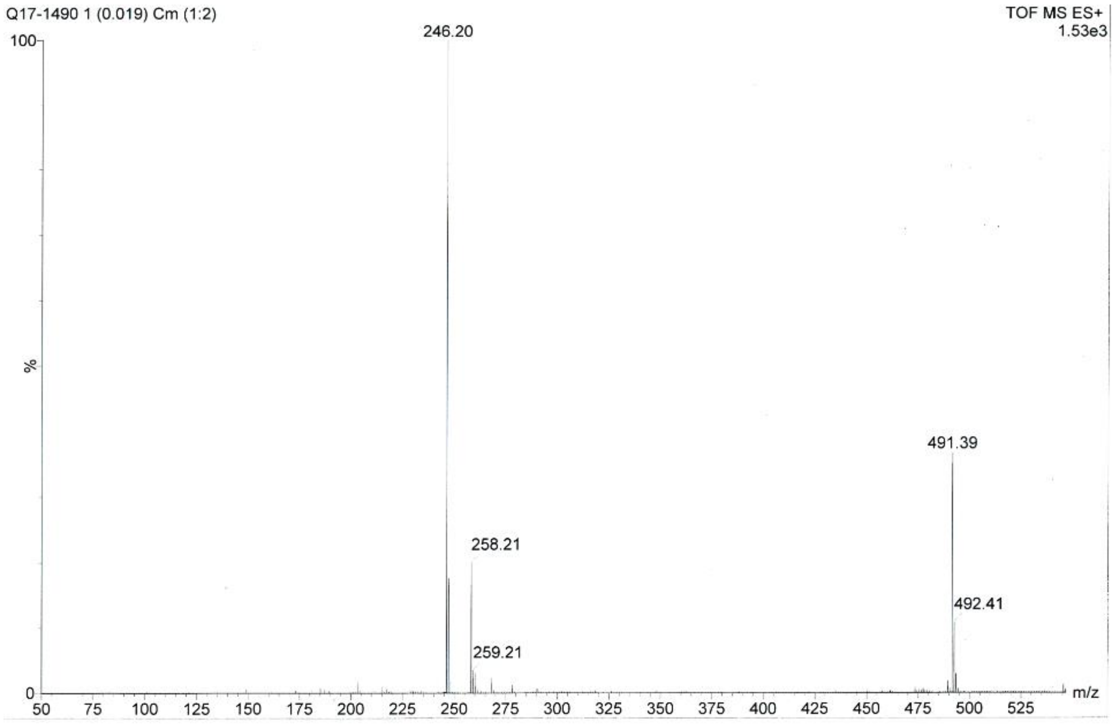

^{1}H NMR and MS data of 15:

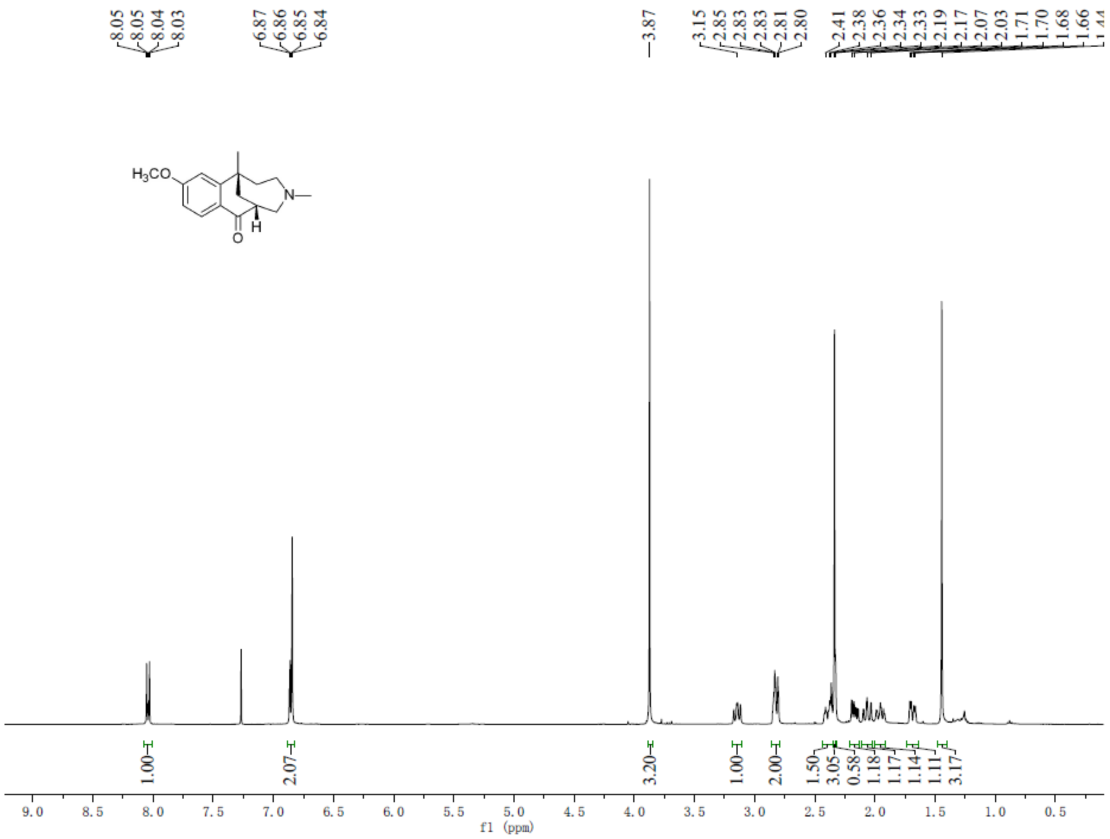

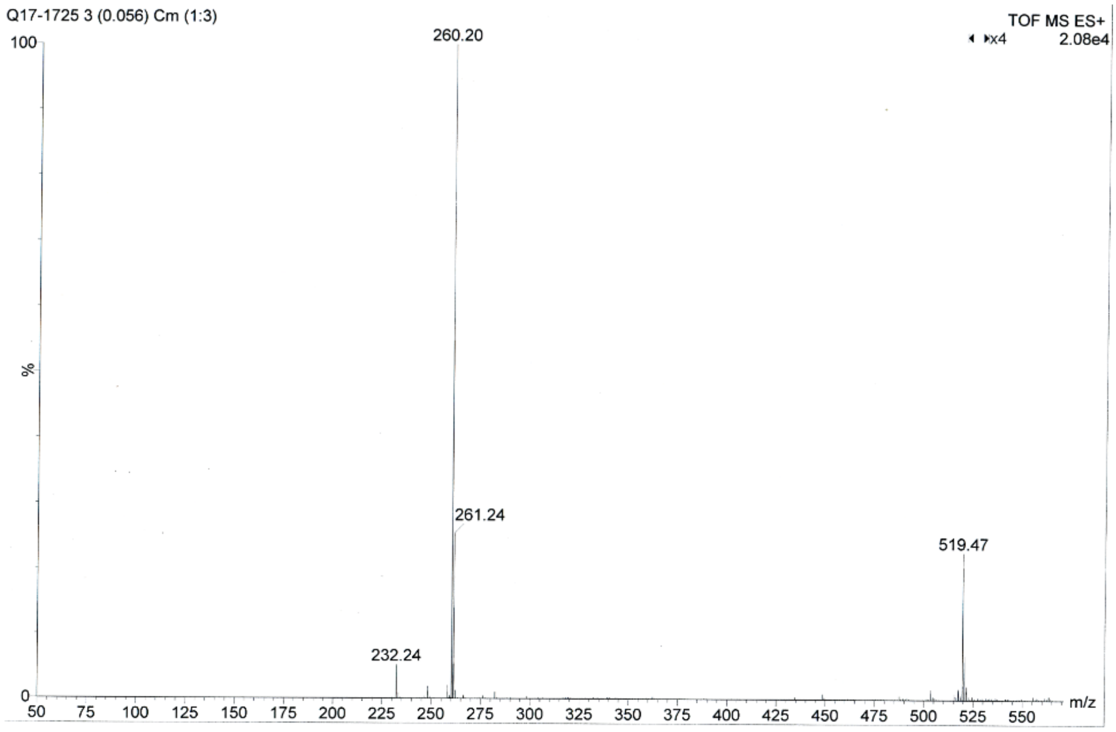

^{1}H NMR and MS data of 8:

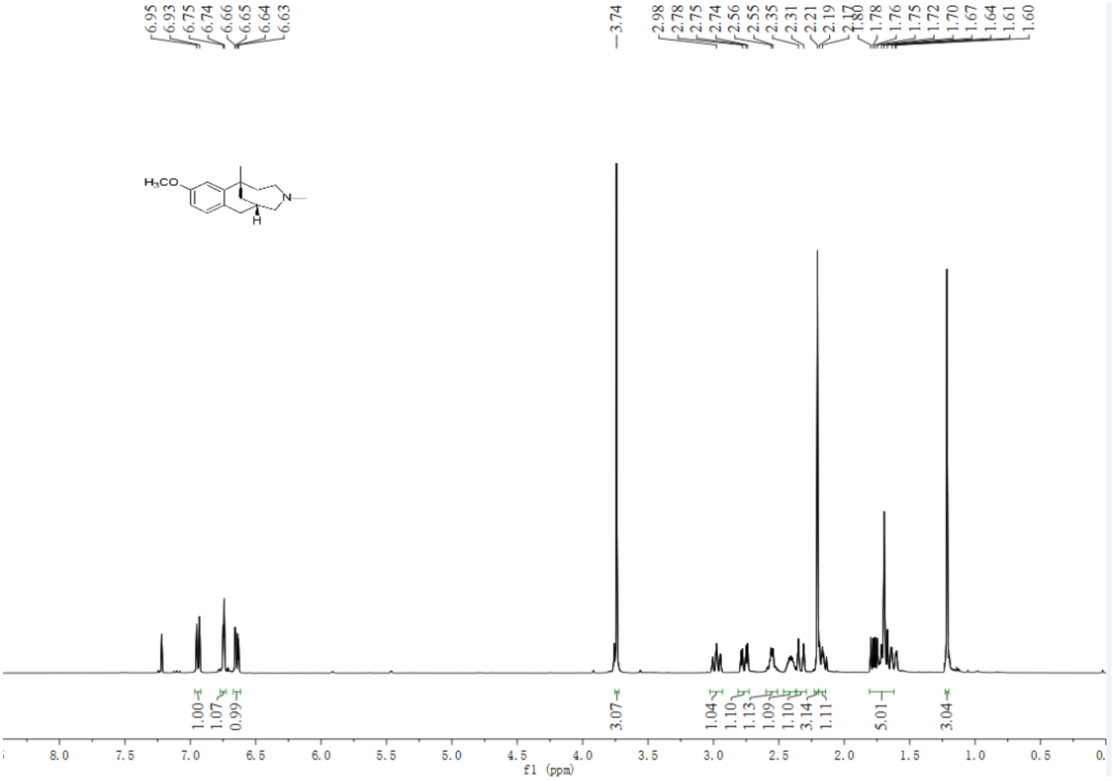

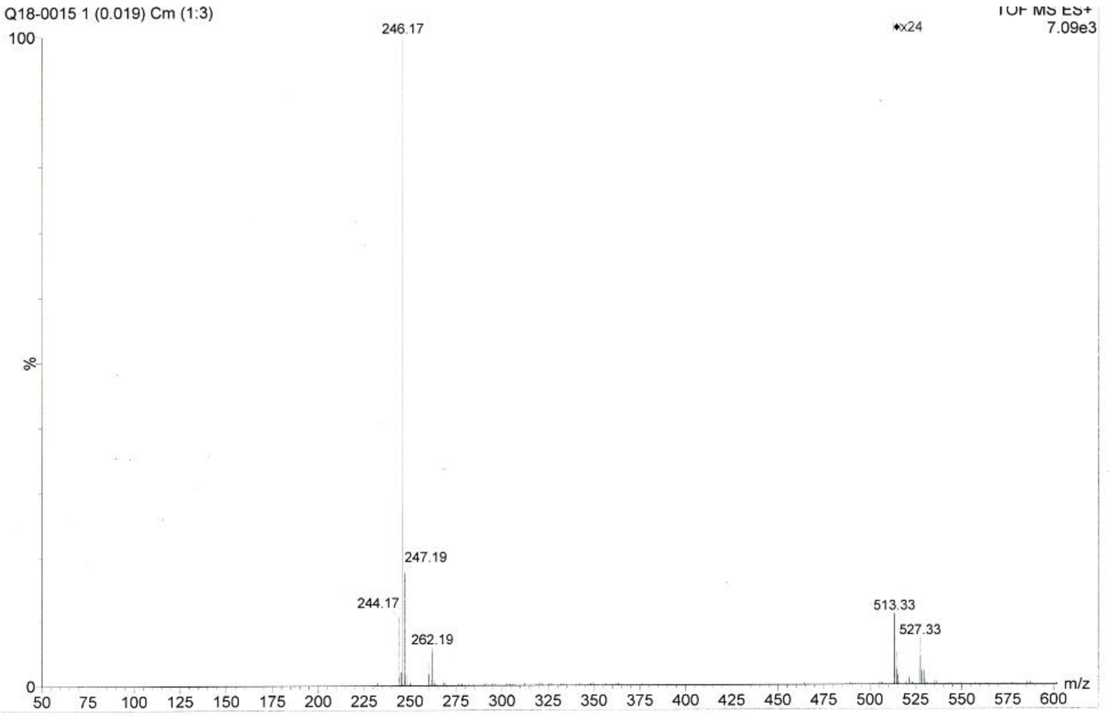

Chiral HPLC chromatograms of 4:

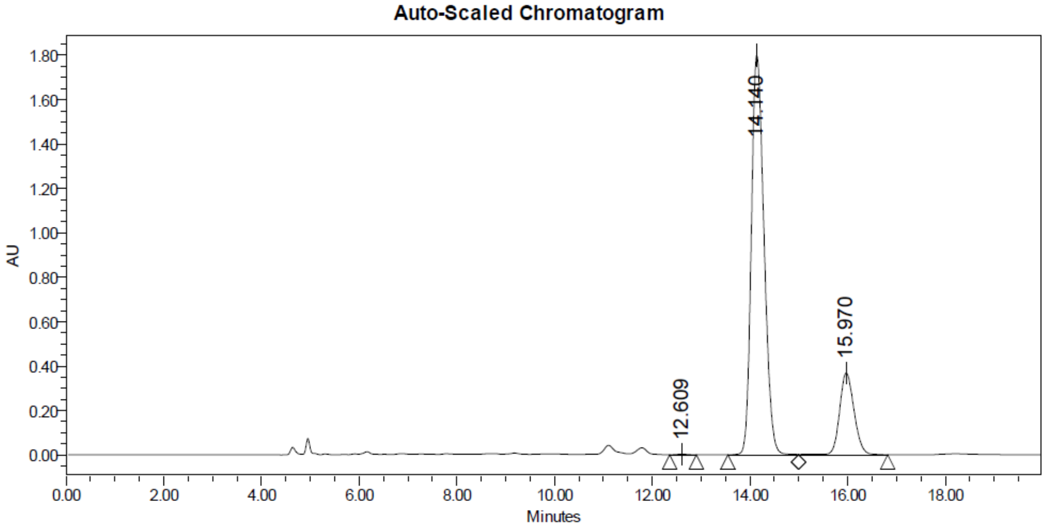

Peak Results

	Name	RT	Area	Height	% Area	USP Resolution	USP Tailing
1		12.609	21228	1346	0.05		1.043953e+000
2		14.140	32376946	1797718	81.11	3.372077e+000	1.225841e+000
3		15.970	7518065	368115	18.83	3.556414e+000	1.146943e+000

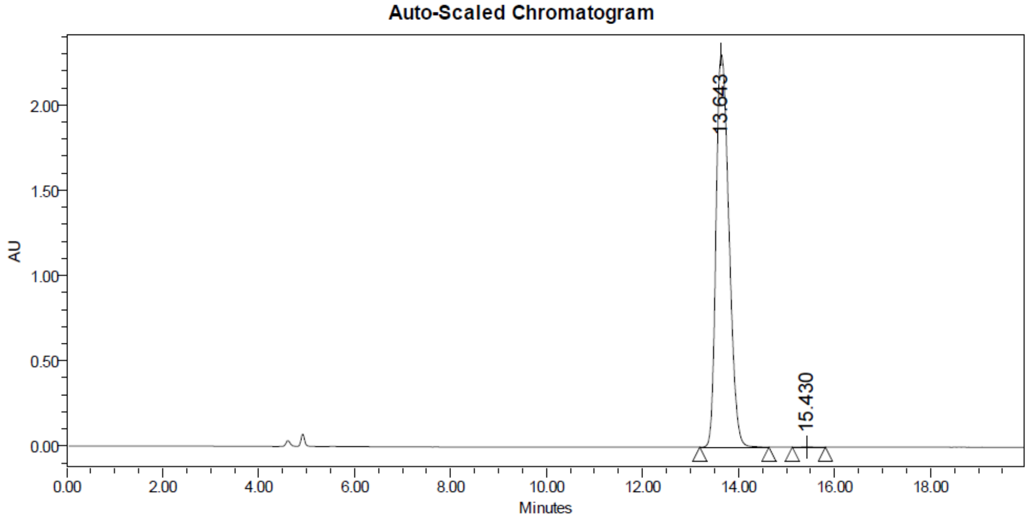

Peak Results

	Name	RT	Area	Height	% Area	USP Resolution	USP Tailing
1		13.643	43093919	2305575	99.92		1.299486e+000
2		15.430	34779	1900	0.08	3.556622e+000	1.083278e+000

^{1}H NMR, MS and HPLC chromatograms of eptazocine hydrobromide (1):

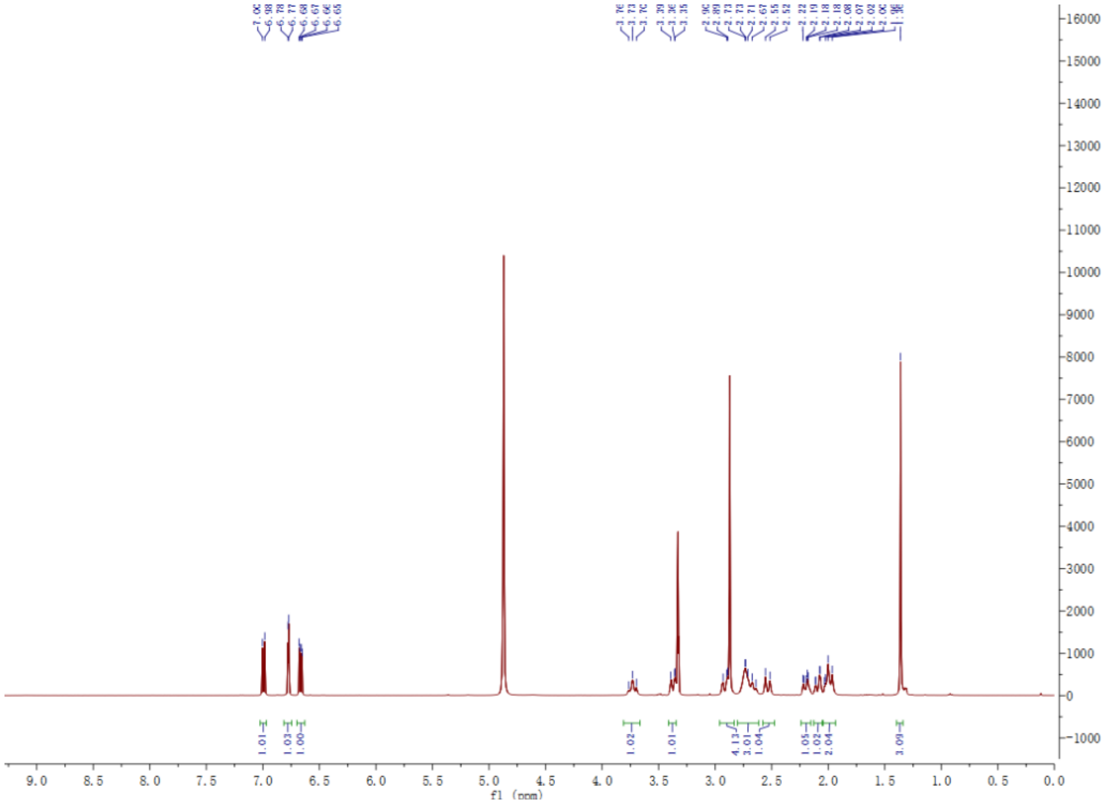

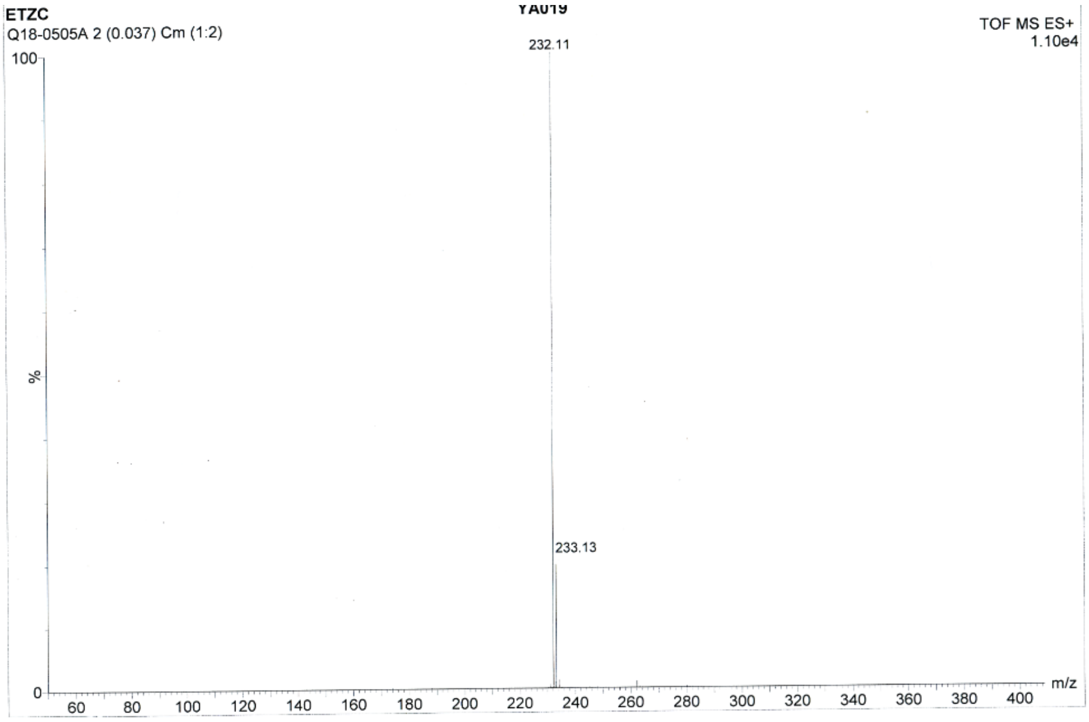

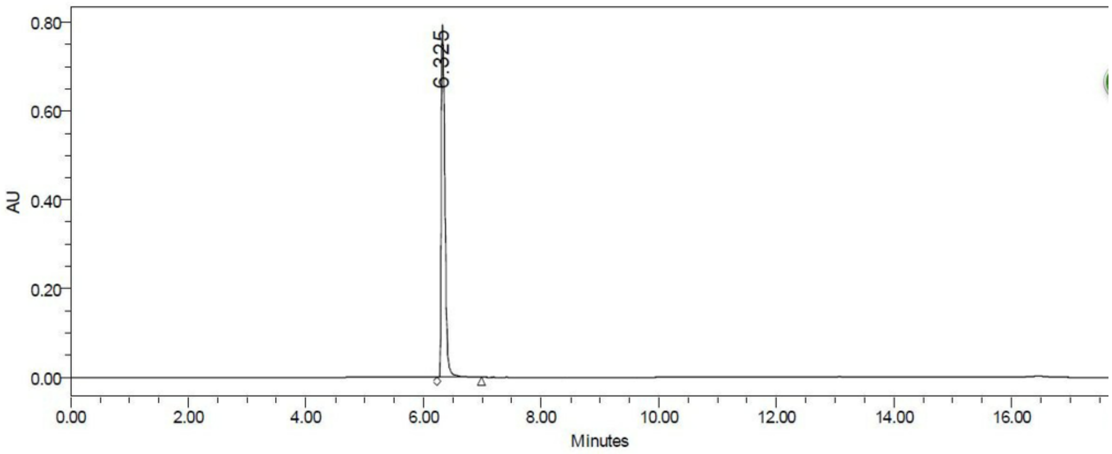

	RT	Area	% Area	Height
1	6.325	3368142	100.00	793764
